# Finite Deformation of Scleral Tissue under Electrical Stimulation: An Arbitrary Lagrangian-Eulerian Finite Element Method

**DOI:** 10.3390/bioengineering10080920

**Published:** 2023-08-03

**Authors:** Jafar Arash Mehr, Hamed Hatami-Marbini

**Affiliations:** Mechanical and Industrial Engineering Department, University of Illinois Chicago, Chicago, IL 60607, USA

**Keywords:** chemo-electro-mechanical model, large deformation, electroactive tissue, sclera

## Abstract

The sclera is considered as the principal load-bearing tissue within the eye. The sclera is negatively charged; thus, it exhibits mechanical response to electrical stimulation. We recently demonstrated the electroactive behavior of sclera by performing experimental measurements that captured the deformation of the tip of scleral strips subjected to electric voltage. We also numerically analyzed the electromechanical response of the tissue using a chemo-electro-mechanical model. In the pre-sent study, we extended our previous work by experimentally characterizing the deformation profile of scleral strips along their length under electrical stimulation. In addition, we improved our previous mathematical model such that it could numerically capture the large deformation of samples. For this purpose, we considered the transient variability of the fixed charge density and the coupling between mechanical and chemo-electrical phenomena. These improvements in-creased the accuracy of the computational model, resulting in a better numerical representation of experimentally measured bending angles.

## 1. Introduction

The sclera is a dense connective tissue constituting more than 80% of the eye’s wall. The sclera is merged with the cornea and extends to the back of the eye. The tough nature of the sclera protects the eye from serious damage like laceration and rupture. The sclera also provides a solid support for extraocular muscles that control the eye movement. The rigidity of the sclera preserves the shape of eye. Furthermore, the opacity of the sclera prevents internal light scattering from affecting the quality of retinal images.

In general, the sclera is a mixture of water (nearly 70% of the weight), collagen, elastin, and interfibrillar proteoglycans (PGs) [1]. PGs play important role in maintaining the structure of sclera tissue [2,3]. They fill the space between collagen and are composed of a core protein with glycosaminoglycan (GAG) side chains. GAGs are found in the extracellular matrix of soft tissues such as the cornea and sclera, determining their hydration, elasticity, resiliency, and integrity [4,5,6,7]. The most important types of GAGs include chondroitin sulfate, dermatan sulfate, and keratan sulfate [8]. GAGs are negatively charged because of carboxyl ${COO}^{-}$ and sulfate ${SO}_{3}^{-}$ groups in their polymer network [9]. In addition to the electrostatic repulsive force among GAGs, their presence inside the sclera affects the distribution of mobile ions because of the electroneutrality condition, which creates a swelling tendency by absorbing liquid from the surrounding medium. Thus, the sclera behaves as a natural biological polyelectrolyte gel and shows mechanical deformation under an external electrical stimulation [10,11].

We recently demonstrated the electroactive bending mechanical response of scleral tissue to the external electrical field [12]. This electromechanical response occurs because of the time-dependent alteration of ion concentration inside the tissue and the surrounding solution [12,13], which leads to the formation of differential osmotic pressure at the interfaces between the tissue and solution [14,15,16,17]. In our previous studies, we characterized scleral electromechanical behavior under an electrical field in terms of its bending angle, defined as the deviation of the tip of samples with respect to its original location. Although, this method has commonly been used to investigate the deformation of electroactive hydrogels in the literature [16,17,18,19,20,21], it does not provide an accurate estimate of the trajectory and profile of the bending deformation of samples. One of objectives of the present study was to address this drawback by better quantifying the profile of bending deformation. For this purpose, we measured bending angles corresponding to different points over the length of scleral strips.

The previous numerical model for the simulation of the transient deformation of scleral strips under electrical stimulation mainly used a one-way coupling between chemo-electrical and mechanical fields without considering the changes in fixed charge concentration [12,13]. However, when the deformation is large, both the transient variation in fixed charge density and the coupling between mechanical and chemo-electrical computational domains need to be considered for accurate numerical prediction of scleral electroactive response. Previous mass transport and theory of porous media models used the assumption of small deformation [22,23,24]. Thus, they were unable to capture the effect of large movement of the electroactive hydrogel domain on the distribution of mobile ions inside the solution [25,26]. In large deformation problems, the movement of the hydrogel domain inside the surrounding solution needs to be considered, when solving the PNP system, for an accurate estimate of mobile ion concentration. To better capture the interaction between mechanical, electrical, and chemical fields, the present work developed a novel numerical model in which the moving mesh technique was used to update the geometry of computational domains as a function of time. This numerical model was used to analyze experimental measurements of the electroactive behavior of the sclera. The integrated numerical and experimental methodology of this research provided a better understanding of the electroactive response of the sclera.

## 2. Materials and Methods

### 2.1. Experiments

**Figure 1:**
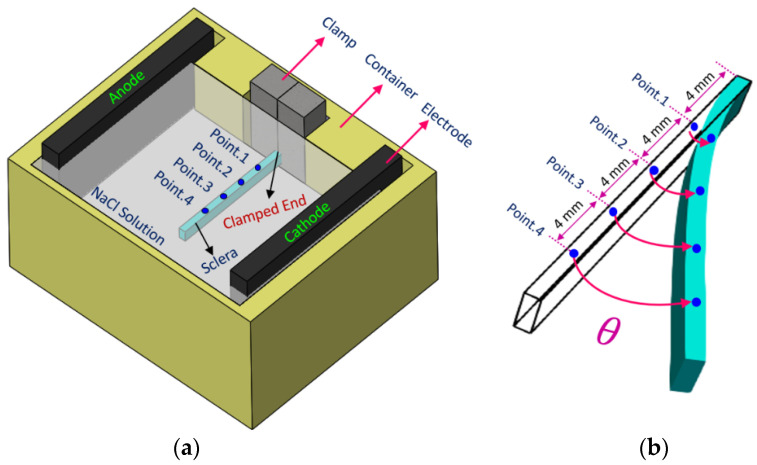
(**a**) Schematic illustration of the target points on a scleral strip. (**b**) Deformation (i.e., bending) of a scleral strip after stimulation by an external electrical field; the location of the target points and bending angles *θ* at each point are shown.

The experimental setup was similar to our previous work with minor modifications [12,13]. First, porcine scleral strips with dimensions of 20 mm×2.5 mm were cut in the superior–inferior direction. After drying them in a desiccator for 24 h, they were immersed in 0.15 M NaCl solution until they reached their constant swelling ratio of ≅2.0 [27]. Then, samples were mounted into the experimental setup between two electrodes at 5 cm apart. A DC power supply was used to apply the electrical voltage and a high-resolution camera was used to monitor the displacement of the sample within 60 s of electrical stimulation. In order to characterize the profile of deformation of strips along their length, we marked four different points (i.e., target points) with approximate distances of 4 mm (point 1), 8 mm (point 2), 12 mm (point 3), and 16 mm (point 4) from the clamped end of scleral strips. Figure 1 schematically shows the location of target points on a scleral sample mounted in the experimental setup.

### 2.2. Numerical Model

#### 2.2.1. Calculation Domain

**Figure 2:**
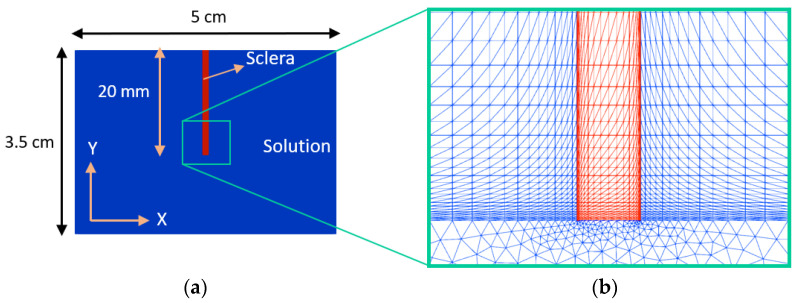
(**a**) The numerical domains consisting of the sclera and solution and (**b**) the respective finite element mesh.

A 2D geometry was created with dimensions consistent with the experimental setup including the chamber (3.5 cm × 5 cm) and the scleral strips (20 mm × 1.3 mm; length × thickness); Figure 2. A structured mesh and an unstructured mesh using triangular Lagrange elements were generated for the area covering the length of samples (0.015 m≤y≤0.035 m) and the lower portion of the solution (0≤y<0.015 m), respectively. We used around 4300 and 1600 finite elements for meshing the solution domain and scleral domain, respectively. The density of elements was increased at the vicinity of sclera–solution interfaces in order to capture the jump in the concentration of the mobile ions [12,13,22,28].

#### 2.2.2. Constitutive Equations

The chemo-electrical fields are represented by the coupled nonlinear Poisson–Nernst–Planck equations, as widely described in other studies [12,13,22].

The Nernst–Planck (NP) equation is expressed as follows:

(1) $$\frac{\partial c_{i}}{\partial t}+\nabla .\left(-D\nabla c_{i}-\mu zFc_{i}\nabla \emptyset \right)=0\;\;\;\;\;\;\;$$

where $c_{i}$ is the concentration of the ith mobile species, D is the diffusion constant, μ is the mobility of ionic species (μ=D/RT), R is the universal gas constant, *T* is the temperature, z is the valence of ions, F is the Faraday constant, and ∅ is the electric potential. The Poisson equation is given as follows:

(2) $$\nabla^{2}\emptyset =-\frac{F}{\varepsilon_{0}\varepsilon_{r}}\left(z_{Na}c_{Na}+z_{Cl}c_{Cl}+z_{Fixed}c_{Fixed}\right)\;\;\;\;\;$$

where $\varepsilon_{0}$ is the electrical permittivity of vacuum, $\varepsilon_{r}\;$is the relative permittivity of the medium, $z_{Na}\;$is the valence of the sodium cation, $z_{Cl}$ is the valence of the chloride anion, $z_{Fixed}$ is the valence of the fixed charge in hydrogel, $c_{Na}$ is the concentration of sodium cation, $c_{Cl}\;$is the concentration of chloride anion, and$\;c_{Fixed}\;$is the concentration of fixed charges in the sclera stemming from the existence of GAGs.

The equation of equilibrium describes the deformation of the samples due to electrical stimulation:

(3) $$\sigma_{ij,j}+f_{i}=\frac{\partial^{2}u_{i}}{\partial t^{2}}\;\;\;\;\;\;$$

where $\sigma_{ij}$, $u_{i}$, and $f_{i}$ correspond to stress, displacement, and body force components, respectively.

#### 2.2.3. Body Force

The differential osmotic pressure is defined as follows [12,13,26,29]:

(4) $$∆\pi =RT\sum_{i{=Na}^{+},\;\;{Cl}^{-}}\left[{{(c}_{i})}_{Sclera}-{{(c}_{i})}_{Solution}\right]$$

**Figure 3:**
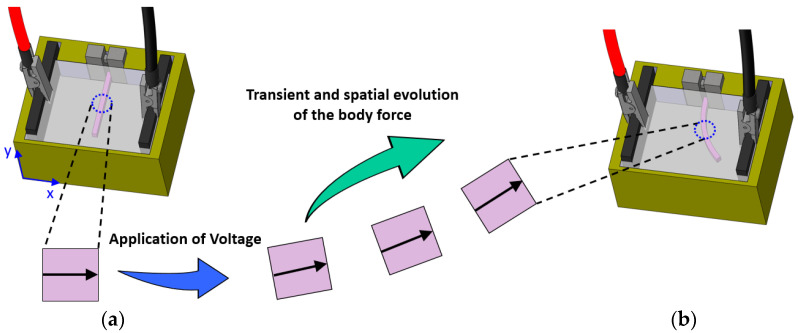
Illustration of the deformation of the sclera domain inside NaCl solution. A scleral strip inside the NaCl solution (**a**) before and (**b**) after the application of the electrical voltage. Black arrows correspond to the vector defining the gradient of body force at an arbitrary distance from the clamped end of the scleral strip and how it evolves in time and space after voltage application.

The body force f correlates chemo-electrical fields described by the PNP system to the mechanical field [12,13]. In our previous work, we neglected the deformation of the scleral domain inside the solution when solving the PNP system. To be more specific, the concentrations of ions were calculated by solving the PNP system when the sclera domain was considered fixed in the NaCl solution. In other words, the gradient of differential osmotic pressure was calculated at every longitudinal distance from the clamped end of the sclera strip along a vector perpendicular to the scleral length, Figure 3, i.e., this gradient was in the horizontal direction (*x* direction). However, when the deformation of the sclera is large, the displacement of the scleral domain inside the solution cannot be neglected when solving the PNP system. This is because this movement causes the gradient of the osmotic pressure to exist not only in the horizontal direction, but also in the vertical direction (*y* direction). As a consequence, body force has two different components in the *x* and *y* directions; Figure 3.

The components of body force are defined as follows:

(5) $$f=\left[\begin{array}{c} \nabla_{x}\left(∆{\tilde{\pi }}_{1}-∆{\tilde{\pi }}_{2}\right)\\ \nabla_{y}\left(∆{\tilde{\pi }}_{1}-∆{\tilde{\pi }}_{2}\right) \end{array}\right]\;\;\;\;\;\;$$

In the above equation, $∆{\tilde{\pi }}_{1}$ and $∆{\tilde{\pi }}_{2}$ are normalized differential osmotic pressure ($∆\tilde{\pi }=∆\pi /RT$) at the anode side and cathode side, respectively.

In our previous work [12,13], body force was uniform over the length of strips with only one component in the *x* direction. This means that the effect of the deformation of the sclera domain inside the solution domain was not considered when calculating the concentrations of mobile species. However, when the scleral deformation is large, i.e., it moves and rotates inside the solution domain in a way that the degree of the rotation significantly increases from the clamped end to the free end, the components of the body force cannot be considered uniform over the length of scleral strips. Here, in each time step, we considered several equally distanced points along the length of the samples and calculated the components of the body force at these points. Then, we defined third-order polynomials $\;f_{x}=\alpha_{1}y^{3}+\alpha_{2}y^{2}+\alpha_{3}y+\alpha_{4}$ and ${\;f}_{y}=\beta_{1}y^{3}+\beta_{2}y^{2}+\beta_{3}y+\beta_{4}$ corresponding to the components of body force in the *x* and *y* directions. Coefficients $\alpha_{i}$ and $\beta_{i}$ were calculated by fitting these polynomials to the calculated body force at target points. The calculation of constants was performed using the differential evolution algorithm [30].

#### 2.2.4. Transient Change in Fixed Charge Density (FCD)

When the sclera deforms and bends towards the cathode because of electrical actuation, a transient volume change occurs in the tissue that in turn reduces the FCD inside the sample. Thus, it is required to calculate the sample volume change and update the FCD in each time step. The new volume of the sample is given as follows [31]:

(6) $$\frac{V_{current}}{V_{initial}}=\left|\nabla u+I\right|\;\;\;\;\;\;$$

where I is the identity tensor; ∇u is the gradient of the displacement field; and $V_{current}$ and $V_{initial}$ are the current and initial volume of samples, respectively. The FCD at the current time is given by the following:

(7) $${\left(FCD\right)}_{current}=\frac{{\left(FCD\right)}_{initial}V_{initial}}{V_{current}}$$

#### 2.2.5. Moving Mesh Implementation

The arbitrary Lagrangian-Eulerian (ALE) method was used [32] to consider the effect of scleral deformation in solving the PNP system. This moving mesh technique was used in order to preserve the quality of the elements at the interfaces of the sclera and solution, which was necessary for calculation of the sharp gradient of mobile ion concentrations at these boundaries. The mesh was generated in GMSH [33] and the numerical model was solved using open-source finite element package FEniCS [34].

#### 2.2.6. Constants and Initial and Boundary Conditions

The constants used in the numerical model are the same as those presented in our previous study [12]. The initial concentrations of sodium and chloride ions were set to 150 mol/m^3^ inside the solution domain. The concentration of mobile species inside the sclera domain was calculated using the Donnan equation [13,35,36]. In addition, 5 V and −5 V were applied on the left and right boundaries of the solution (i.e., anode and cathode, respectively). Zero displacement in the *x* and *y* directions was applied at the top of the sample (i.e., clamped end). Furthermore, the electrical stimulation was applied for 60 s with 0.01 s time steps.

#### 2.2.7. Fitting the Numerical Model

We used the numerical model for the analysis of our experimental data and found model parameters, i.e., the FCD and the Young’s modulus E, using the differential evolution algorithm [13,30,37]. We used a mutation factor of 0.5 and a crossover parameter of 0.9 in the algorithm [38]. The cost function was defined as follows:

(8) $$Cost\;Function=\sqrt{\sum_{k=1}^{4}\sum_{i=0}^{n}{\left(\theta_{ik,exp}-\theta_{ik,num}\right)}^{2}}\;\;\;\;\;\;\;$$

where k represents target points 1, 2, 3, and 4 and *i* corresponds to time = 10 s, 20 s, 30 s, 40 s, 50 s, and 60 s. Furthermore, $\theta_{ik,exp}$ and $\theta_{ik,num}$ denote the experimental and numerical bending angles, respectively, of the *k*th target point at time *i*. We selected the range of FCD equal to 5 mM≤FCD≤30 mM to cover a reasonably wide range for FCD. The posterior scleral FCD has been reported to be around 10 mM [12]. The elasticity modulus of the porcine sclera has been reported to be in the range from 0.1 MPa to 2 MPa. Thus, we selected 0.1 MPa≤E≤2 MPa in the optimization procedure.

## 3. Results

### 3.1. Experimental Results

**Figure 4:**
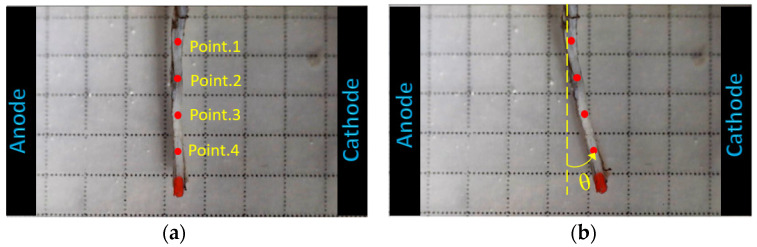
Illustration of the target points on the sclera sample (**a**) before and (**b**) 60 s after the application of 10 V of electrical stimulation.

As the focus of this study was to investigate the profile of deformation over the length of samples, we used a single voltage equal to 10 V that was applied for 60 s. The location of target points 1–4 is illustrated in Figure 1. Figure 4 demonstrates the electroactive bending response of a sclera strip obtained from the experiments after 1 min of applying 10 V of electrical voltage to the electrodes.

**Figure 5:**
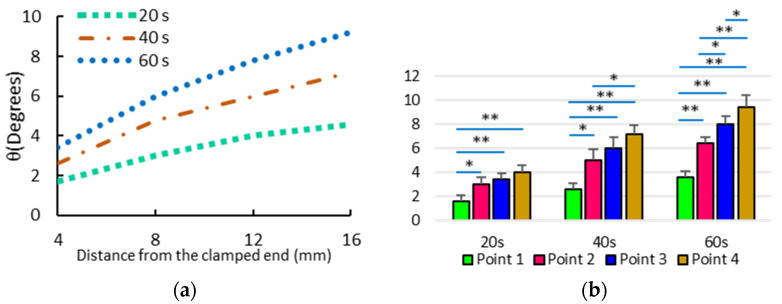
(**a**) The average measured bending angles versus distance from the clamped end of the strips under 10 V of electrical stimulation at different times. (**b**) The bending angles obtained after 20 s, 40 s, and 60 s corresponding to the different points. The significant effect of the longitudinal distance of target points on the determination of the corresponding bending angle after 60 s was found by conducting one-way ANOVA. *p*-values ≤ 0.05 and *p*-values ≤ 0.001 are shown with * and **, respectively.

Figure 5 represents the measured bending angles with respect to the distance from the clamped end of samples at different times. The results were obtained from six different scleral strips stimulated in 0.15 M NaCl under 10 V for 60 s. One-way ANOVA statistical analysis was performed in order to characterize the significance of the distance from the clamped end, in the measured bending angle after 60 s. *p*-value ≤0.05 indicated significant difference.

### 3.2. Bending Angles at the Target Points

**Figure 6:**
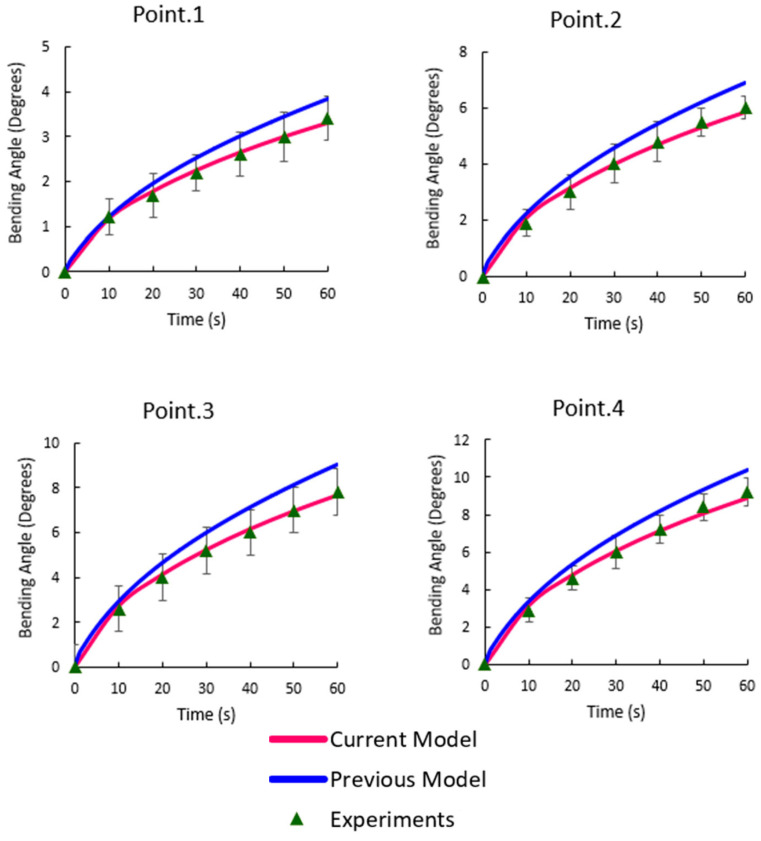
Comparison between the experimentally measured bending angles at points 1–4 and the numerical results (current model vs. previous model).

The experimental bending angle at each target point is presented in Figure 6. The performance of our previous and current numerical model in capturing the experimental measurements is also shown in this figure.

### 3.3. Concentrations of Mobile Ions

**Figure 7:**
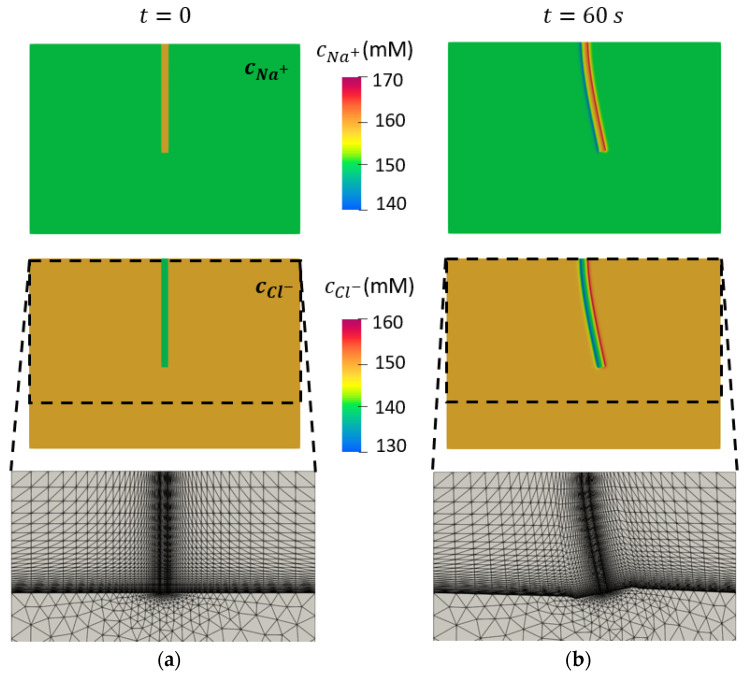
Numerical results for $c_{{Na}^{+}}$ and $c_{{Cl}^{-}}$. The left boundary is the anode and the right boundary is the cathode. (**a**) Results before the application of the electrical voltage are shown. (**b**) Results are presented after application of 10 V for 1 min. The finite element mesh is shown at the bottom before and at the end of electrical stimulation.

The average values for the FCD and Young’s modulus, providing the best fit to the experimental data, were 16.13 mM and 0.52 MPa, respectively. These values were used in numerical studies of the following sections. The concentration of mobile ions before and after the application of 10 V of electrical stimulation for 1 min is shown in Figure 7.

**Figure 8:**
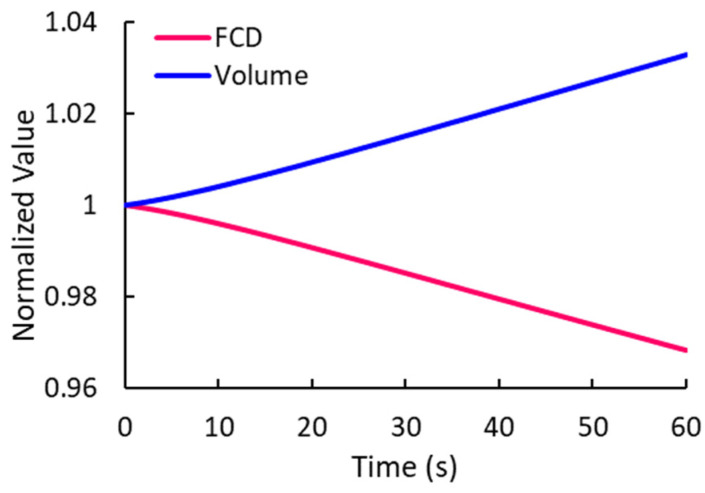
Transient change in the FCD and volume of the hydrogel. The values are normalized to their initial values.

The variation of the normalized FCD and volume over time is presented in Figure 8.

### 3.4. Spatial Distributions of the Mobile Ions at the Target Points

**Figure 9:**
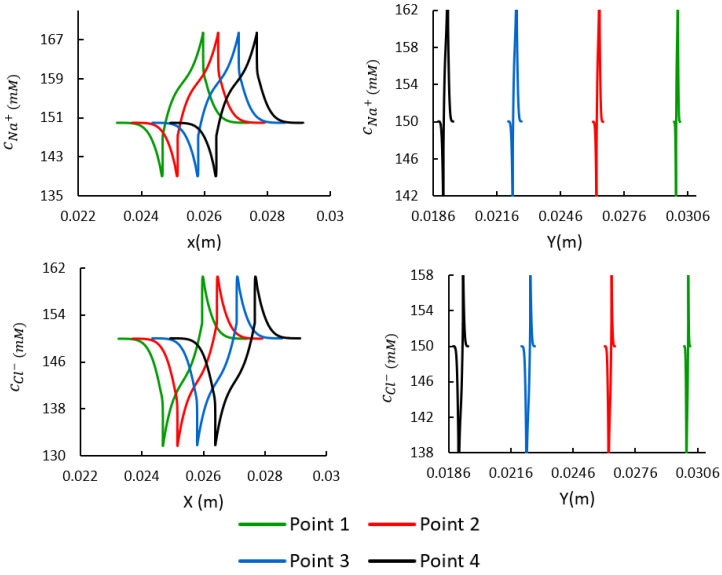
Comparison of the distributions of mobile ions at target points in the *x* and *y* directions.

This section compares the numerical results for different target points (i.e., points 1–4). The distributions of the concentration of mobile ions (i.e., $c_{{Na}^{+}}$ and $c_{{Cl}^{-}}$) in the *x* and *y* directions are shown in Figure 9.

**Figure 10:**
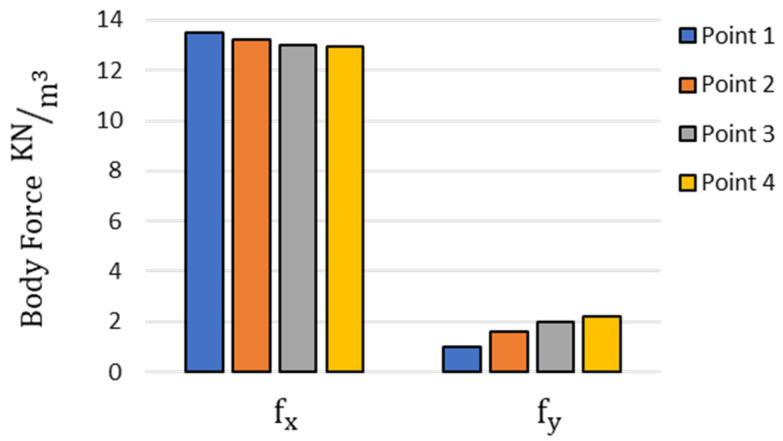
Comparison of the variation in the components of body force generated inside the hydrogel domain after 60 s of electrical stimulation at different longitudinal distances along the length of the sclera strip.

The variation in components of body force at different distances from the clamped end of the sclera strip after 60 s of electrical stimulation is presented in Figure 10.

## 4. Discussion

The electromechanical bending response of an electroactive hydrogel has been extensively characterized using the bending angle of samples, in which this parameter is defined as the deviation of their tip with respect to their initial straight configuration [18,19,20,39]. Although this method to some extent gives insight into the deformation of the samples, it neglects the exact deformation profile along the length of strips. One of the main purposes of this study was to shed more light on the profile of deformation of the scleral strips when they undergo electrical stimulation inside an electrolyte solution. In the present study, we selected multiple points along the length of strips to track the bending angle of each point as a function of time. The location of these target points is shown in Figure 1. The scleral strips, similar to the behavior of polyanionic hydrogels, bent toward the cathode after being triggered by the external electrical voltage. In other words, each point of the sclera strips moved inside the solution domain as the sclera was deformed. When the deformation is large, the transient spatial variation of domains interacting with each other affects the gradient of ion concentrations at the boundaries of the sclera and solution. We used an ALE moving mesh technique in order to smoothly move and update the required finite element mesh to solve the chemo-electrical fields based on the displacements obtained from solving the mechanical field. This method was developed in order to take care of the existing coupling between the computational domains in different physics. It should be noted that other numerical models proposed for the simulation of mechanical response of the electroactive hydrogels neglected this important issue, as they were developed based on the assumption of small deformation of hydrogels [13,22,25,29,40].

We measured the bending angles for different target points in our experiments when the scleral strips underwent 10 V of electrical stimulation with the purpose of giving more scrutiny to the profile of deformation along the length. The bending angles of points 1, 2, 3, and 4 after 60 s reached an average of 3.6°, 6.4°, 8°, and 9.4°, respectively, corresponding to the distances of 4 mm, 8 mm, 12 mm, and 16 mm, respectively, from the clamped end of samples (Figure 5). The bending angles of target points (i.e., points 1–4) were presented versus their distances from the clamped end in Figure 5, representing the bending trajectory along the length of samples at different times. According to the statistical analysis, the effect of the longitudinal location in the bending angles became more statistically significant between the target points by prolonging the electrical stimulation (Figure 5b).

In this work, we used the differential evolution optimization algorithm to tune the numerical model against the experimental data. We showed that the present model was able to numerically represent the electromechanical bending response of the sclera. It should be noted that the optimization was conducted based on experimental data measured at several points along the length of samples. This approach provided better estimation of unknowns by feeding more experimental data to the algorithm compared with the case where the data corresponding to only one point were used in the fitting procedure [13].

It was found that, although previous models provided reasonable results to capture the experimental data, they overestimated the bending angles at the points 1–4 (Figure 6). The current model provided a better fit by reducing the gap between the experimental data and numerical estimates. One of the improvements in this model was the consideration of the transient variation in FCD because of the volume change of samples during the electrical stimulation [29,41]. The high sensitivity of the bending behavior of electroactive hydrogels to the content of fixed charges has been confirmed previously [42]. In general, the deformation of samples had a direct relation with the FCD. We found that the FCD gradually decreased in time (Figure 8).

The distributions of mobile ions in the *x* and *y* directions at different points along the length of the sclera strips revealed how the locations of target points change after 60 s of electrical stimulation under 10 V (Figure 9). This indicated how the boundaries of the sclera domain were moved inside the solution domain at different longitudinal distances. Figure 7 shows the distribution of sodium and chloride ions. The movement of the mesh was performed in a way that the quality of finite elements was preserved at the interfaces between the sclera and solution domain. This was indispensable to be able to capture the sharp gradient of the concentration of mobiles species at boundaries of the sclera and solution domain [12,13].

Furthermore, we were able to capture the component of body force in the *y* direction ($f_{y}$), which was not present in previous numerical models [12,13]. In previous models, body force had only one component in the *x* direction as the sclera domain was considered fixed in the solution domain in the calculation of ion concentrations. When comparing different components of body force (i.e., $f_{x}$ and $f_{y}$), it was found that the distribution of body force was not uniform over the length of strips. Although $f_{x}$ decreased by moving from the clamped end to the free end, $f_{y}$ increased, as depicted in Figure 10. It should be noted that the large rotation of the sclera domain inside the solution was considered in the current model for calculating mobile ion concentrations. As a matter of fact, the rotation of the vector defining the body force at each longitudinal distance increased from the clamped end to the free end. This explains the larger contribution of the vertical component of body force and lower contribution of the horizontal component.

The present work proposed an experimental and computational framework that found the mechanical properties of the tissue considering its chemical and electrical properties. The numerical model was used to analyze experimental data in order to estimate the stiffness and fixed charge density of the sclera. However, this work had several limitations. For example, the 2D linear elastic material model is a simplistic assumption that was used here to speed up the necessary simulations. The primary objectives of the present work were to capture the profile of deformation experimentally and to develop a numerical model using a moving finite element mesh. In general, a nonlinear hyperelastic material model is suitable for simulation of the mechanical response of a soft tissue [43]. We recently used an anisotropic hyperelastic material model to simulate the electroactive bending response of the sclera tissue [13]. This model can represent the nonlinear and anisotropic material properties of the sclera and should ideally have been used. However, this model required the expansion and implementation of the current model in 3D, which would have increased the computational cost significantly. We are currently working on this computational framework and present the summary of the necessary constitutive equations in the Appendix A. In addition, porcine sclera samples were only used in the experimental study. Although the water content, histology, and organization of collagen fibers in human and porcine sclera are similar, there are certain differences that could cause them to show a different electromechanical response. For example, porcine sclera is thicker compared with human sclera [44]. This study could be extended to human samples in the future. Despite its limitations, this study gave a deeper insight into the electroactive mechanical response of sclera tissue by accurate experimental representation of the scleral deformation profile. Furthermore, it developed a numerical model based on the dynamic variation of the FCD and computational domains for the first time. This numerical model successfully captured the experimental data and significantly increased the reliability of the numerical analysis.

## 5. Conclusions

We created a large deformation chemo-electro-mechanical model for the simulation of the electroactive bending response of the sclera. We considered coupling between different physics by taking advantage of the ALE moving mesh strategy. We considered the spatial nonuniform variation in body force along the length of samples in both directions. Furthermore, we considered transient and spatial alteration in concentrations of ionic species. We measured the deformation data at four different points over the length of sclera strips rather than only at the tip of samples. Thus, we provided an accurate experimental estimate of their longitudinal profile of the deformation under electrical stimulation. We used the numerical model to analyze experimental measurements and find the stiffness and negative fixed charge density inside the tissue. The present numerical model demonstrated higher accuracy compared with previous models in terms of capturing the bending behavior of the sclera tissue under electrical stimulation.

## Data Availability

Requests about the data presented in this study should be sent to the corresponding author.

## Appendix A

The strain energy function for a hyperelastic material model that is able to consider the anisotropic orientation and concentration of collagen fibers in the sclera tissue is presented here [13,38].

(A1) $$\tilde{W}=F_{1}\left({\tilde{I}}_{1}\right)+\int_{\theta_{P}-\frac{\pi }{2}}^{\theta_{P}+\frac{\pi }{2}}P\left(\theta \right)F_{2}\left(\tilde{\lambda }\left[\theta \right]\right)d\theta +\frac{K}{2}{\left(ln\left(J\right)\right)}^{2}\;\;\;\;\;\;\;$$

In the above equation, K is the bulk modulus; $\theta_{P}$ determines the orientation of the fibers; J is the determinant of deformation gradient tensor; and $P\left(\theta \right)$ is the distribution function of collagen fibers, i.e.,

(A2) $$P\left(\theta \right)=\frac{1}{\pi I_{0}(k)}\;\exp\left(k_{f}\cos\left(2\left(\theta -\theta_{p}\right)\right)\right)$$

where

(A3) $$I_{0}\left(k\right)=\frac{1}{\pi }\int_{0}^{\pi }e^{\left(k_{f}\;cos\left(x\right)\right)}\;\;\;\;\;\;$$

is the Bessel function of the first kind. In the above equation, $k_{f}$ is the fiber concentration factor controlling collagen fiber alignment along a preferred fiber orientation $\theta_{p}$. The fourth invariant $I_{4}(\theta )$ is defined as

(A4) $$I_{4}\left(\theta \right)=a_{0}\left(\theta \right)\cdot C\cdot a_{0}\left(\theta \right)=\lambda^{2}\left(\theta \right)$$

where $a_{0}\left(\theta \right)$ is the unit vector representing the direction of collagen fibers and C is the right Cauchy deformation tensor. In Equation (A1), the strain energy of the matrix is given as follows:

(A5) $$F_{1}\left({\tilde{I}}_{1}\right)=c_{1}\left({\tilde{I}}_{1}-3\right)$$

where ${\tilde{I}}_{1}$ corresponds to the first invariant of $\tilde{C}$, which is the deviatoric part of the right Cauchy deformation tensor, and $c_{1}$ is the first Mooney–Rivlin material coefficient. In addition, the energy contribution of collagen fibers is expressed as follows:

(A6) $$F_{2}\left(\tilde{\lambda }\right)=c_{3}\left(e^{-c_{4}}\left(Ei\left(c_{4}\tilde{\lambda }\right)-Ei\left(c_{4}\right)\right)-ln\left(\tilde{\lambda }\right)\right)$$

where *Ei* is the exponential integral function, $c_{3}$ is the exponential fiber stress coefficient, and $c_{4}$ is the rate of uncrimping collagen fibers. After some manipulation, the Cauchy stress tensor is found as follows:

(A7) $$\sigma =pI+\frac{2}{J}\left[c_{1}\left(\tilde{B}-\frac{1}{3}{\tilde{I}}_{1}I\right)+\int_{\theta_{P}-\frac{\pi }{2}}^{\theta_{P}+\frac{\pi }{2}}P\left(\theta ,k\right){\tilde{W}}_{4}{\tilde{I}}_{4}\left(\theta \right)\left(a_{0}\left(\theta \right)⨂a_{0}\left(\theta \right)-\frac{1}{3}I\right)d\theta \right]$$

where

(A8) $${\tilde{W}}_{4}\left(\theta \right)=\frac{1}{2{\tilde{\lambda }}^{2}\left(\theta \right)}c_{3}\left[e^{c_{4}\left(\tilde{\lambda }\left(\theta \right)-1\right)}-1\right]$$

and *p* and $\tilde{B}$ are the hydrostatic pressure and deviatoric part of the left Cauchy deformation tensor, respectively.
